# Cell-free DNA profiling of metastatic prostate cancer reveals microsatellite instability, structural rearrangements and clonal hematopoiesis

**DOI:** 10.1186/s13073-018-0595-5

**Published:** 2018-11-21

**Authors:** Markus Mayrhofer, Bram De Laere, Tom Whitington, Peter Van Oyen, Christophe Ghysel, Jozef Ampe, Piet Ost, Wim Demey, Lucien Hoekx, Dirk Schrijvers, Barbara Brouwers, Willem Lybaert, Els Everaert, Daan De Maeseneer, Michiel Strijbos, Alain Bols, Karen Fransis, Steffi Oeyen, Pieter-Jan van Dam, Gert Van den Eynden, Annemie Rutten, Markus Aly, Tobias Nordström, Steven Van Laere, Mattias Rantalainen, Prabhakar Rajan, Lars Egevad, Anders Ullén, Jeffrey Yachnin, Luc Dirix, Henrik Grönberg, Johan Lindberg

**Affiliations:** 1 Department of Medical Epidemiology and Biostatistics, Karolinska Institutet, Stockholm, Sweden; 2 Centre for Oncological Research, University of Antwerp, Antwerp, Belgium; 3 Department of Urology, AZ Sint-Jan, Brugge, Belgium; 4 Department of Radiation Oncology, Ghent University Hospital, Ghent, Belgium; 5 Department of Oncology, AZ KLINA, Brasschaat, Belgium; 6 Department of Urology, Antwerp University Hospital, Antwerp, Belgium; 7 Department of Oncology, ZNA Middelheim, Antwerp, Belgium; 8 Department of Oncology, AZ Sint-Jan, Brugge, Belgium; 9 Department of Oncology, AZ Nikolaas, Sint-Niklaas, Belgium; 10 Department of Oncology, AZ Sint-Lucas, Brugge, Belgium; 11 Department of Pathology, GZA Hospitals Sint-Augustinus, Antwerp, Belgium; 12 Department of Oncology, GZA Hospitals Sint-Augustinus, Antwerp, Belgium; 13 Centre for Molecular Oncology, Barts Cancer Institute, Cancer Research UK Barts Centre, Queen Mary University of London, London, UK; 14 Department of Oncology-Pathology, Karolinska Institutet and University Hospital, Stockholm, Sweden; 15 Department of Medical Epidemiology and Biostatistics, Science for Life Laboratory, Karolinska Institutet, Stockholm, Sweden

**Keywords:** Circulating tumor DNA, Metastatic prostate cancer, Microsatellite instability, Structural rearrangement, Clonal hematopoiesis

## Abstract

**Background:**

There are multiple existing and emerging therapeutic avenues for metastatic prostate cancer, with a common denominator, which is the need for predictive biomarkers. Circulating tumor DNA (ctDNA) has the potential to cost-efficiently accelerate precision medicine trials to improve clinical efficacy and diminish costs and toxicity. However, comprehensive ctDNA profiling in metastatic prostate cancer to date has been limited.

**Methods:**

A combination of targeted and low-pass whole genome sequencing was performed on plasma cell-free DNA and matched white blood cell germline DNA in 364 blood samples from 217 metastatic prostate cancer patients.

**Results:**

ctDNA was detected in 85.9% of baseline samples, correlated to line of therapy and was mirrored by circulating tumor cell enumeration of synchronous blood samples. Comprehensive profiling of the androgen receptor (AR) revealed a continuous increase in the fraction of patients with intra-*AR* structural variation, from 15.4% during first-line metastatic castration-resistant prostate cancer therapy to 45.2% in fourth line, indicating a continuous evolution of AR during the course of the disease. Patients displayed frequent alterations in DNA repair deficiency genes (18.0%). Additionally, the microsatellite instability phenotype was identified in 3.81% of eligible samples (≥ 0.1 ctDNA fraction). Sequencing of non-repetitive intronic and exonic regions of *PTEN*, *RB1*, and *TP53* detected biallelic inactivation in 47.5%, 20.3%, and 44.1% of samples with ≥ 0.2 ctDNA fraction, respectively. Only one patient carried a clonal high-impact variant without a detectable second hit. Intronic high-impact structural variation was twice as common as exonic mutations in *PTEN* and RB1. Finally, 14.6% of patients presented false positive variants due to clonal hematopoiesis, commonly ignored in commercially available assays.

**Conclusions:**

ctDNA profiles appear to mirror the genomic landscape of metastatic prostate cancer tissue and may cost-efficiently provide somatic information in clinical trials designed to identify predictive biomarkers. However, intronic sequencing of the interrogated tumor suppressors challenges the ubiquitous focus on coding regions and is vital, together with profiling of synchronous white blood cells, to minimize erroneous assignments which in turn may confound results and impede true associations in clinical trials.

**Electronic supplementary material:**

The online version of this article (10.1186/s13073-018-0595-5) contains supplementary material, which is available to authorized users.

## Background

Prostate cancer is the most commonly detected male cancer in Europe and the third major cause of cancer-related death among men [1]. Although the majority of metastatic hormone-naïve prostate cancers (mHNPCs) demonstrate a reliable response to initial androgen deprivation therapy which targets AR signaling, progression to a castration-resistant state is inevitable. However, the treatment landscape for metastatic castration-resistant prostate cancer (mCRPC) is evolving with the recent approval of several new drugs translating to an increased overall survival [2–6]. Multiple additional avenues exist as genomic profiling of metastatic tissue revealed that the majority of mCRPC patients harbor clinically relevant alterations beyond the AR signaling pathway [7].

The most promising non-approved treatment avenue in metastatic prostate cancers (mPCs) exploits synthetic lethality in treating homologous recombination-deficient cancers with poly (ADP-ribose) polymerase (PARP) inhibitors [8]. Approximately one fifth of mCRPC carry mutations in DNA repair genes [7]. However, the mutational signatures of biallelic inactivation are heterogeneous between different DNA repair genes [9], and future studies are therefore needed to determine which genes are associated with a response to PARP inhibition. Approximately 3% of mPC are driven by microsatellite instability (MSI) [7, 10]. Pembrolizumab recently became the first drug to be approved by the U.S. Food and Drug Administration based on the MSI phenotype, irrespective of tumor type [11]. Although checkpoint blockade did not confer a survival advantage as compared with placebo for chemotherapy-relapsed mCRPC [12], anecdotal cases have been reported to display partial or complete responses [10, 13–15].

The emergence of additional drugs, both towards common and rare mPC phenotypes such as PTEN-deficient [16, 17] and neuroendocrine cancers [18], raises questions of how to efficiently translate the multitude of treatment options to improved patient outcomes. The genomic heterogeneity of mCRPC [7] and, in turn, the low response rates of currently approved drugs [2–5, 19, 20] argue for the urgent need of predictive biomarkers. Ineffective trial-and-error decisions inevitably lead to unnecessary side effects and unsustainable costs [21]. The AR splice variant 7 (AR-V7) [22] demonstrated promising results as a negative response biomarker for androgen receptor signaling inhibitors. However, follow-up studies have been unable to validate the initial clear-cut finding [23, 24], and although AR-V7 is clearly prognostic, clinical implementation remains debated due to (1) lack of treatment options for AR-V7-positive patients and (2) lack of data from a prospective clinical trial demonstrating the predictive power of AR-V7 for treatment selection [25]. Recently, Scher and colleagues demonstrated increased survival for AR-V7-positive patients receiving taxanes in a blinded retrospective multicenter study [26]. However, AR-V7 assay positivity and taxane therapy were both correlated to tumor burden, obscuring interpretation and further highlighting the need for a prospective randomized clinical trial to determine if AR-V7 can be applied as a predictive biomarker [27].

The lack of predictive biomarkers is in part due to the difficulty of obtaining temporally matched metastatic tissue as the majority of mPCs metastasize to the bone. Multiple studies on the acquisition of tumor tissue with or without direct image guidance report a range of success rates [28–31]. A recent effort, focusing on methodological improvements, obtained > 20% cell content in the majority of bone biopsies [32]. Circulating tumor DNA is a viable alternative to metastatic tissue with demonstrated high fractions of ctDNA [33–36] enabling sensitive detection of somatic variation, and direct comparisons to metastatic tissue have revealed high concordance [33, 37, 38]. Circulating tumor DNA has several advantages as sampling through simple blood draws is fast, cost efficient, and without side effects and allows for longitudinal monitoring and the detection of multiple resistance alleles during therapy [38, 39].

Although ctDNA has the potential to accelerate biomarker-driven trials in mPC, several questions remain unanswered, e.g. if it is possible to detect MSI directly from liquid biopsies and how ctDNA fractions correlate to line of therapy. The ctDNA fraction determines the sensitivity to detect somatic variation which in turn has consequences for the design of prospective biomarker studies relying on liquid biopsies. Here, we present a retrospective analysis of 217 cases and 364 blood samples covering the entire spectrum of mPC. The purpose of this study was to gather information relevant for future liquid biopsy-driven biomarker studies with a focus on (1) how ctDNA fractions vary from mHNPC to end stage castration-resistant disease; (2) a rationale for how to treat samples with low ctDNA fraction; (3) the relative impact of different types of somatic variation, affecting the sequencing strategy; (4) the detection of potentially predictive biomarkers; (5) and finally, how clonal expansions in the hematopoietic stem cells [40–43] impact liquid biopsy profiling.

## Methods

A detailed description of the methods is provided in Additional file 1: Supplemental methods. In brief, mPC patients were recruited in an all-comer cohort (*n* = 217), ranging from hormone-naïve to castration-resistant, between 2014 and 2017 with histologically confirmed prostate adenocarcinoma (Table 1). Blood samples (*n* = 364) were collected at the start of a new line of therapy (defined as “baseline” sample) or during a particular systemic therapy (defined as “follow-up” sample). The complete study cohort (*n* = 217) encompassed patients recruited as part of the ProBio (Prostate Biomarkers) study (*n* = 72, Stockholm, Sweden) and abiraterone- or enzalutamide-treated patients (*n* = 145) recruited to the CORE-ARV-CTC study (Antwerp, Belgium). The original purpose of the CORE-ARV-CTC cohort was to investigate if profiling androgen receptor splice variants in circulating tumor cells (CTCs) may predict response to enzalutamide and abiraterone treatment. The result of this analysis together with somatic alterations in AR and TP53 is presented in a separate manuscript [24]. Additionally, anonymous healthy donor blood (*n* = 36) was collected (Stockholm, Sweden). The study was conducted in accordance with the Declaration of Helsinki, after approval was acquired by ethical committees in Belgium (Antwerp University Hospital, registration number: B300201524217) and Sweden (Karolinska University Hospital, registration number: 2016/101-32). All patients provided a written informed consent document. Plasma was enriched from 2 × 10 ml (ProBio patients) or 4–5 ml (CORE-ARV-CTC patients) EDTA blood and stored at − 80 °C within the same working day, allowing for high-quality ctDNA profiling [44]. Germline DNA was extracted from leftover EDTA blood. In addition, for 340 out of 364 ctDNA-analyzed blood samples, an additional blood sample was collected in a CellSave tube and shipped to the GZA Sint-Augustinus (Antwerp, Belgium) for CTC enumeration within 72 h on the FDA-cleared CellSearch platform (Menarini Silicon Biosystems, Italy), as previously described [45]. Upon isolation, 0.1–50 ng of cell-free DNA (cfDNA) and 50 ng of germline DNA were used to create the sequencing libraries (ThruPLEX Plasma-seq kit, Rubicon Genomics, USA). Cell-free DNA profiling was performed with a mix of low-pass whole genome sequencing and hybridization-capture targeted sequencing (SeqCap EZ system, Roche Nimblegen, USA). Germline samples were only processed by the latter. The capture designs and their targeted regions are described in Additional file 2: Table S1.

**Table 1:** Clinical characteristics describing the study participants

	Number	Percentage
Patients	211*	100
Age at first sampling year, mean ± SD	73.0 ± 8.91	
Tumor stage at diagnosis		
T1/2	44	20.9
T3/4	50	23.7
M1	74	35.1
Node-positive	15	7.1
Not specified	28	13.3
Gleason score at diagnosis		
≤ 7	72	34.1
8–10	108	51.2
Not specified	31	14.7
Primary treatment		
ADT (± RT/CT)	125	59.2
Radical Px (± RT)	61	28.9
Radical Px + ADT	7	3.3
Other	18	8.5
Metastatic burden at first sampling		
LN only	31	14.7
Bone only	73	34.6
Bone and LN	61	28.9
Visceral and bone and/or LN	34	16.1
Not specified	12	5.7
Stage at first sampling (all patients, *n* = 217)		
mHNPC	23	10.6
mHSPC1	6	2.8
mCRPC	188	86.6

*Six individuals declined access to medical records

Low-level processing of the sequencing data was performed as previously described [36] and analyzed as described in detail in Additional file 1: Supplemental methods, which allowed for identification of pathogenic germline variants, copy-number alterations (CNAs), small mutations, and structural variation in unique regions in the human genome commonly mutated in prostate cancer (bioinformatic tools and settings are summarized in Additional file 3: Table S2). The detection of somatic alterations, which was copy-number adjusted, allowed the estimation of tumor burden (ctDNA fraction, the fraction of cfDNA molecules originating from the cancer cells). Where somatic CNA analysis suggested a higher tumor burden, it was instead calculated from the CNA profile. Additionally, the incorporation of microsatellites in the comprehensive capture design allowed the evaluation of microsatellite instability (MSI). Finally, the analysis of patient-matched cfDNA and germline DNA samples, compared to a merged file of all healthy donor blood samples as a control, allowed the interrogation of clonal hematopoiesis. Statistical analysis was performed in R (v3.3.2) [46].

## Results

### Liquid biopsy profiling of metastatic prostate cancer

Comprehensive cfDNA profiling was performed on 217 mPC patients (Table 1). Single nucleotide variants, copy-number alterations (CNAs), and genomic structural rearrangements were interrogated using a combination of in-solution hybridization capture-based and low-pass whole genome sequencing. An evolution of capture designs was applied as the project progressed, from a pan-cancer to a prostate-specific approach to cost-efficiently maximize the information content (Additional file 2: Table S1). The comprehensive designs were aimed at progression samples with high tumor burden whereas the smaller designs were tailored for cost-efficient deep sequencing. However, to increase the sensitivity to detect, e.g., intronic structural variation in *AR*, the majority of samples were processed with both a comprehensive and a smaller design (Additional file 4: Table S3). The data was subsequently merged before variant calling. The average coverage, taking merging into account, was 814× (interquartile range 251–965) for cfDNA and 445× (interquartile range 371–533) for germline DNA. Data from all samples is presented here, where the number of relevant samples is described for each section (Additional file 5: Table S4). In total, 364 plasma samples from 217 mPC patients were profiled. Circulating tumor cell enumeration using the CellSearch platform was performed from synchronous blood draws on 340 of the 364 plasma samples.

### Circulating tumor DNA fraction correlation to line of therapy

**Fig. 1:**
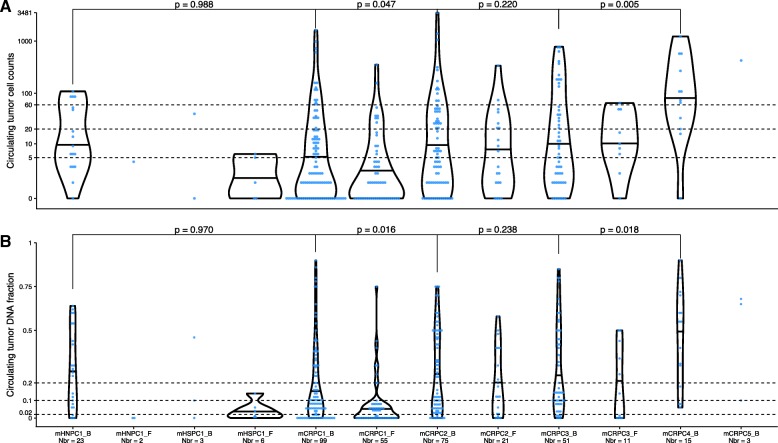
Tumor burden at different lines of therapy. **a** Violin plot of the circulating tumor cell counts per 7.5 ml of blood using the CellSearch platform partitioned according to line of therapy. The black horizontal lines within the violin plots denote the median of the density estimate. Blue points represent the circulating tumor cell counts in individual blood samples. A one-sided Wilcoxon rank sum test was applied to investigate if the baseline samples of, for example, mCRPC1 had lower tumor burden than mCRPC2. *Y*-axis: log10 transformed circulating tumor cell counts. *X*-axis: line of therapy. **b** as **a** but for circulating tumor DNA fraction. *Y*-axis: circulating tumor DNA fraction. In total, 364 blood samples from 217 cases are displayed here; however, only 340/364 had a successful circulating tumor cell count. The dashed lines at 0.02, 0.10, and 0.20 denote the cutoffs to reliably detect point mutations, loss of heterozygosity, and homozygous deletions, respectively. Abbreviations: mHNPC[number], metastatic hormone naïve prostate cancer and line of therapy; mHSPC[number], metastatic hormone-sensitive prostate cancer and line of therapy; mCRPC[number], metastatic castration-resistant prostate cancer and line of therapy; _B, baseline, blood samples collected at start of a new systemic therapy; _F, follow-up, blood samples collected during a systemic therapy; Nbr, number of cell-free DNA samples profiled in each category

Circulating tumor DNA was detected in the majority of baseline samples (85.9%, Additional file 6: Figure S1). However, as the fraction of ctDNA in the cfDNA influences the sensitivity to detect somatic variation, we investigated if tumor burden correlated to line of therapy and blood draw timing (Additional file 7: Table S5). Comparing baseline ctDNA fractions at different lines of therapy, a significant increase was present between first- and second-line mCRPC and third- and fourth-line mCRPC (Fig. 1). The ctDNA fractions were significantly lower, comparing baseline and follow-up samples for mHNPC and first-line mCRPC (Additional file 6: Figure S2). The differences were not statistically significant for later lines of therapy. The CTC counts were correlated to the ctDNA fraction estimate (rho = 0.7, *p* < 0.0001) (Additional file 6: Figure S3) and mimicked the ctDNA pattern in relation to line of therapy (Fig. 1, Additional file 6: Figure S2).

### Detection of microsatellite instability from cell-free DNA

**Fig. 2:**
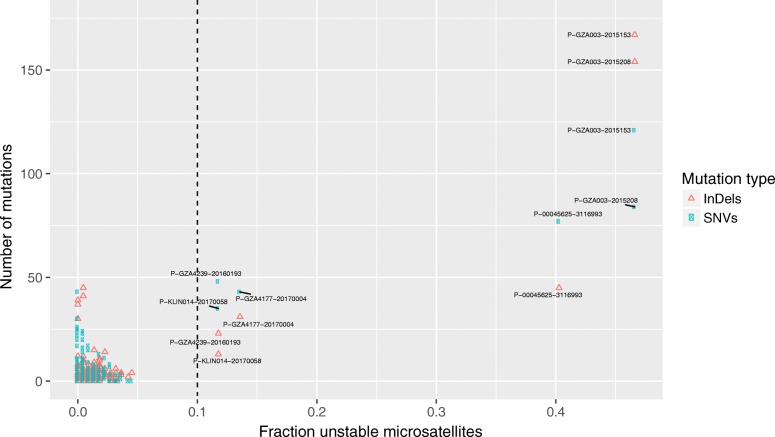
Detection of microsatellite instability from cell-free DNA. Microsatellite unstable tumors were identified from 121 samples (105 unique patients) with ≥ 0.1 circulating tumor DNA fraction by plotting the number of mutations (*Y*-axis, including intronic and synonymous variants) versus the fraction of unstable microsatellite loci (*X*-axis). Indels and single nucleotide variants are kept separate for each sample, colored according to the right legend. The dashed vertical line at 0.10 fraction unstable microsatellites denotes the cutoff to reliably detect microsatellite instability. Two separate cell-free DNA samples were profiled for P-GZA003, and both demonstrated microsatellite instability. Note that although individual P-KLIN014, sample 20170058, demonstrated > 0.1 fraction unstable microsatellite loci, it was classified as microsatellite stable. The sample had a high circulating tumor DNA fraction (0.80), lacked an increase in number of mutations and displayed high copy-number burden, indicative of a chromosomal instability phenotype

Microsatellites were targeted and sequenced to enable MSI-phenotype detection directly from cfDNA (Additional file 2: Table S1 and Additional file 5: Table S4). Using an in-house cohort of colorectal cancer samples (Additional file 6: Figure S4), in silico dilution with germline DNA demonstrated 100% sensitivity and 99% specificity to detect MSI at 10% tumor purity and 10% unstable microsatellites with the mSINGS algorithm [47]. Applying mSINGS on ≥ 10% ctDNA fraction samples identified four cases with MSI out of 105 investigated (Fig. 2). The proportion of MSI-positive cases detected from ctDNA is concordant with a previous study based on whole-exome sequencing of metastatic tissue samples (two-sided Fisher’s exact test: *p* = 0.721) [7].

### Intronic sequencing of key tumor suppressors and biallelic inactivation

**Fig. 3:**
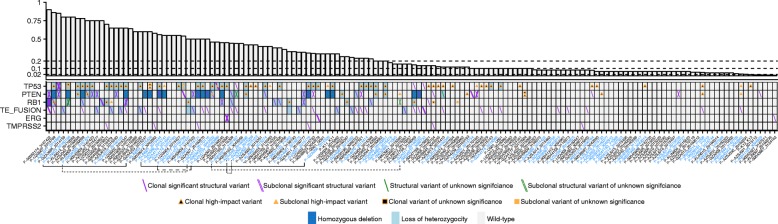
Exonic and intronic profiling of circulating tumor DNA. The non-repetitive sequence was captured for the entire gene body of *TP53*, *PTEN*, and *RB1* in 165 cell-free DNA samples from 135 men. The somatic variants found in the 152 cell-free DNA samples from 124 men with detectable circulating tumor DNA are shown here. *TMPRSS2-ERG* gene fusions or structural rearrangements in *TMPRSS2* or *ERG* are also shown. The upper panel displays the circulating tumor DNA fraction. The dashed lines at 0.02, 0.10, and 0.20 denote the cutoffs to reliably detect point mutations, loss of heterozygosity, and homozygous deletions, respectively. Bottom panel, heatmap of the somatic alterations detected from circulating tumor DNA profiling. Type of alteration is coded according to the bottom legend. For visualization purposes, up to two mutations or structural variants (forward and backslashes) are displayed per patient. Triangles and boxes represent single nucleotide variants and indels, respectively. Subclonal mutations are defined as having an allele frequency < 1/4 of the circulating tumor DNA fraction. The same definition was applied to structural variants after median allele frequency adjustment with respect to the mutations. Synonymous point mutations are not displayed here. Variants of unknown significance are non-synonymous single nucleotide variants outside hotspots, not annotated as pathogenic in variant databases. Structural variants of unknown significance are, for example, confined to a single intron, without affecting neighboring exons. *X*-axis: cell-free DNA samples sorted according to the circulating tumor DNA fraction. Patients with multiple samples are colored in blue. The asterisk indicates samples with microsatellite instability. Samples described in the main text are connected with lines

Prostate cancer is mainly driven by CNAs, commonly generated through chained structural rearrangements. Chained events cause the majority of *TMPRSS2-ERG* gene fusions [48], also observed in our data (Fig. 3, Additional file 6: Figure S5). To allow detection of structural rearrangements, capture probes were designed towards the non-repetitive intronic and exonic regions of *PTEN*, *RB1*, and *TP53* in the prostate-specific comprehensive design (CP design, Additional file 2: Table S1, Additional file 5: Table S4, Additional file 8: Table S6, Additional file 6: Figure S6). Structural rearrangements, mutations, and copy-number alterations were investigated in 165 cfDNA samples from 135 study participants profiled with the CP design that passed the internal quality control for structural variant calling (Additional file 1: Supplemental methods, Additional file 5: Table S4). Seventy-one samples (71/165, 43.0%) from 59 men (59/135, 43.7%) had a ctDNA fraction ≥ 0.2, where all classes of somatic variation were detectable. Biallelic inactivation, through clonal high-impact variation, occurred in 47.5% (28/59), 20.3% (12/59), and 44.1% (26/59) of patients in *PTEN*, *RB1*, and *TP53*, respectively. After excluding the MSI samples (carrying high-impact passenger mutations in multiple genes), all samples with a clonal high-impact variant also harbored a second event with only one exception: two samples were profiled for patient P-00030277 and both revealed a 392-kb deletion encompassing exon 9–10 of *TP53* without any observable second hit.

### Clonal dynamics during treatment

Subclonal high-impact variation was detected in multiple patients. Both samples of P-GZA006 revealed subclonal *TP53* mutation accompanied by subclonal deletion (Fig. 3). Before the start of abiraterone therapy, sample 20160759 of patient P-GZA4777 carried two subclonal *TP53* mutations (hotspot and frameshift), a subclonal translocation in *PTEN*, and a weak *AR* amplification (Additional file 6: Figure S7A). At progression (sample 20160890), the hotspot *TP53* variant, *PTEN* translocation and the *AR* amplification were undetectable. However, the *TP53* frameshift increased in allele fraction and a new structural deletion in *TP53* was found, in line with *TP53* loss being associated with rapid progression [49]. P-KLIN003 also experienced change in clonal composition during abiraterone therapy (Additional file 6: Figure S7B). At baseline, two *TP53* mutations were detected. After therapy, the two displayed different behavior, decreasing and increasing in allele fraction. The progressing clone also presented with *TP53* loss of heterozygosity and multiple structural variants in *AR*. Patient P-00039325 had high ctDNA fraction despite being on androgen deprivation therapy for 3 weeks. Following docetaxel treatment, P-00039325 progressed after 215 days with a translocation in *BRCA2* and concomitant loss of heterozygosity (Additional file 6: Figure S7C). In addition, an *AR* amplification and intra-*AR* structural variation were detected.

### Continuous evolution of somatic variation in the androgen receptor

**Fig. 4:**
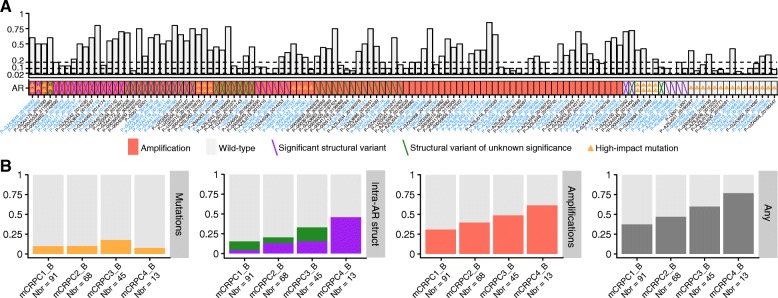
Androgen receptor alterations. Comprehensive profiling of *AR* was performed in 275 cell-free DNA samples from 177 mCRPC patients. **a** The upper panel displays the circulating tumor DNA fraction. The dashed lines at 0.02, 0.10, and 0.20 denote the cutoffs to reliably detect point mutations, loss of heterozygosity, and homozygous deletions, respectively. Bottom panel, heatmap of the mutational landscape detected in the androgen receptor from circulating tumor DNA profiling. Type of alteration is coded according to the bottom legend. For visualization purposes, only samples with an alteration are shown here (126 samples from 89 individuals). Up to two mutations or structural variants (forward and backslashes) are displayed per sample. *X*-axis: cell-free DNA samples sorted according to the number of alterations detected. Patients with multiple samples are colored in blue. The asterisk indicates samples with microsatellite instability. **b** The fraction of patients with alterations in the androgen receptor is categorized by type of alteration and line of therapy. Only high-impact mutations, e.g., hotspot mutations, are shown here. Intra-*AR* structural variation is colored according to the legend in **a**. The rightmost bar plot represents the fraction of patients with any alteration in the androgen receptor. Abbreviations: mCRPC[number], metastatic castration-resistant prostate cancer and line of therapy; _B, baseline; Nbr, number of samples profiled

Comprehensive profiling of *AR*, including intronic sequencing, was performed in 275 mCRPC plasma samples from 177 individuals (Fig. 4a, Additional file 5: Table S4). In total, 45.8% (126/275) of the samples and 50.3% (89/177) of the patients harbored one or more variants in *AR* (high-impact mutation, structural variant, or amplification) in at least one cfDNA sample (Additional file 8: Table S6). Intra-*AR* structural variation was closely correlated to amplification, and only 3/51 patients (P-GZA4045, P-GZA4120, P-U001) carried intra-*AR* structural variation without an accompanying amplification. Structural variation was detected in another three patient samples without amplification (P-AZSJ022, P-KLIN002, P-UZA002), but weak amplifications were found in other samples from the same individuals, taken at other occasions. The fraction of patients with structural variation in *AR* correlated to line of therapy, ranging from 15.4% during first-line mCRPC therapy to 45.2% in the fourth line. Overall, the percentage of individuals with any alteration in *AR* increased from 37.4% in the first line to 76.9% in the fourth line, indicating a continuous evolution of *AR* during the course of the disease (Fig. 4b).

### Alterations in DNA repair deficiency genes

**Fig. 5:**
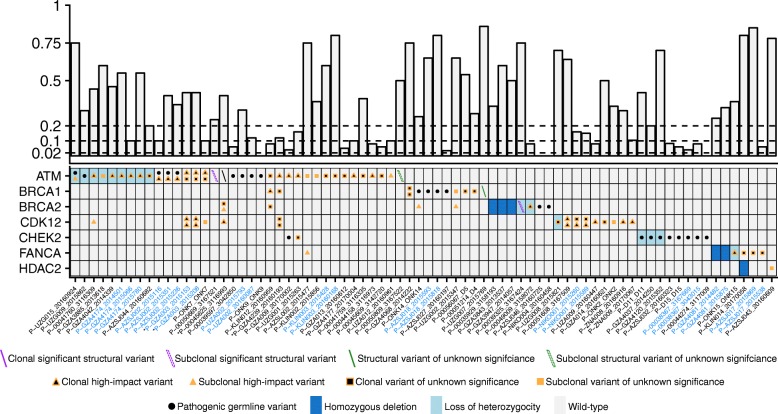
Alterations in genes associated with DNA repair deficiency. The upper panel displays the circulating tumor DNA fraction. The dashed lines at 0.02, 0.10, and 0.20 denote the cutoffs to reliably detect point mutations, loss of heterozygosity, and homozygous deletions, respectively. Bottom panel, heatmap of the mutational landscape detected from circulating tumor DNA profiling of 327 cell-free DNA samples from 217 individuals. For visualization purposes, only the 76 samples with a relevant alteration are shown here. Type of alteration is coded according to the bottom legend. Up to two mutations or structural variants (forward and backslashes) are displayed per patient. Triangles and boxes represent single nucleotide variants and indels, respectively. Subclonal mutations are defined as having an allele frequency <1/4 of the circulating tumor DNA fraction. The same definition was applied to structural variants after median allele frequency adjustment with respect to the mutations. The *BRCA2* structural variant of patient P-00039325, sample 3167424, was classified as borderline subclonal although relevant in the progressing clone after chemohormonal treatment (Additional file 6: Figure S7C). Synonymous point mutations are not displayed here. Variants of unknown significance are non-synonymous single nucleotide variants outside hotspots, not annotated as pathogenic in variant databases. Structural variants of unknown significance are for example confined to a single intron, without affecting neighboring exons. *X*-axis: cell-free DNA samples sorted according to the number of alterations detected in each gene in alphabetical order. Patients with multiple samples are colored in blue. The asterisk indicates samples with microsatellite instability

Genes associated with DNA repair deficiency and commonly mutated in prostate cancer were targeted for mutations and deletions (Additional file 2: Table S1). Sequencing of germline DNA revealed high-impact variants in 8.92% (*ATM*, *BRCA1*, *BRCA2*, and *CHEK2*), similar to recent reports [50–52]. However, only 2/213 (excluding four germline DNA samples that failed processing) carried pathogenic *BRCA2* mutations, significantly less than Pritchard et al. [51] and Mandelker et al. [52] (two-sided Fisher’s exact test: *p* = 0.00329, *p* = 0.00129, respectively; Additional file 9: Table S7). Both reported multiple occurrences of Ashkenazi Jewish founder mutations such as the *BRCA2* p.Ser1982Argfs*22, not observed in this report. Coverage was manually inspected using the integrative genomics viewer [53] which excluded technical causes. This suggests differences in the underlying population demographics. Excluding MSI-positive cases, 18 (8.29%) individuals had somatic biallelic inactivation of a DNA repair gene, whereas 39 (18.0%) had one detectable alteration (Fig. 5, Additional file 8: Table S6). Note, however, that the intronic regions were not targeted in the current version of these capture designs rendering structural variation undetectable, except close to exons or baits designed for CNA purposes.

### Clonal hematopoiesis causes false positive findings

**Fig. 6:**
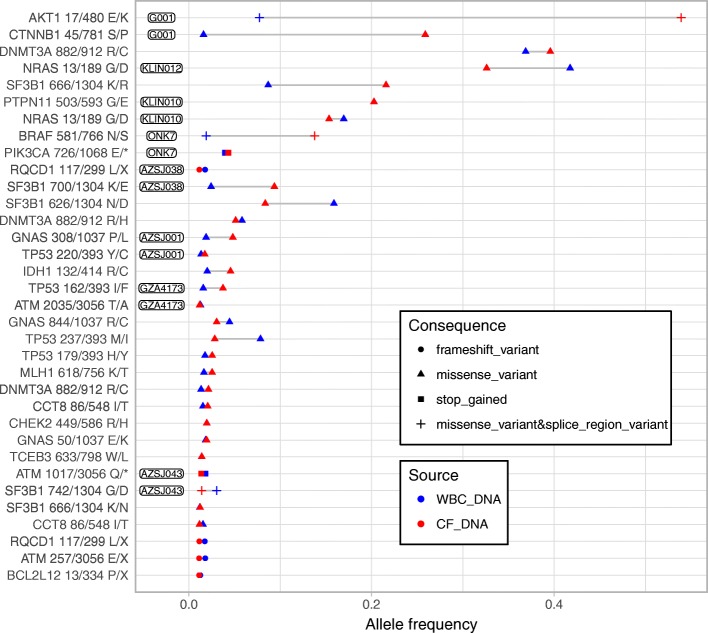
Clonal hematopoiesis. Presence of clonal expansions in the white blood cell compartment was investigated by somatic mutation (single nucleotide variants and indels) analysis. Somatic mutations, supporting existence of clonal hematopoiesis, were identified in germline DNA extracted from white blood cells by using a pool of healthy donor DNA as reference and subsequently validated in cell-free DNA from the same individual. For each mutation, the amino acid position and total number of amino acids are given. Patients with multiple mutations are labeled with sample donor ID. *X*-axis: variant allele frequency. *Y*-axis: individual mutations sorted according to allele frequency in white blood cells and individual. The inset legend explains the type and the source of each variant

Aberrant blood cell populations [40–43] have the potential to confound ctDNA mutational profiles when performed without matched blood DNA as a control. To assess the potential impact and prevalence of genetically aberrant blood cell expansions in our cohort, we investigated copy-number and mutational data for indications of aberrations present in both cfDNA and white blood cell (WBC) DNA. We observed, in separate patients, four cases of large arm-level copy-number alteration (chr 11, 13, and 20) in WBC with coverage ratio and single nucleotide polymorphism allele ratio suggesting a cellularity between 40 and 65% and a focal CCND1 amplification with coverage ratio 1.57, and all were similarly observed in the cfDNA. Putative hematopoietic somatic point mutations were interrogated in WBC DNA using pooled healthy donor DNA as control and excluding variants exceeding 25% allele ratio and outside known somatic hotspots as likely germline. Thirty-seven protein-altering variants were observed in another 29 patients and could be validated in patient-matched cfDNA, including hotspot mutations in *AKT1*, *BRAF*, *CTNNB1*, *DNMT3A*, *NRAS*, *SF3B1*, and *TP53* (Fig. 6). In summary, 40 false positive variants in 31 patients (14.6%) would have been included in ctDNA mutational profiles if matched WBC had not also been sequenced.

## Discussion

Genomics-guided therapy selection is arguably the most promising avenue to remedy trial-and-error treatment decisions and the accelerating costs of drugs [21]. However, the utility of tumor profiling is currently limited in mPC due to the lack of validated predictive biomarkers. Liquid biopsies have the potential to act as a tissue substitute and cost-efficiently accelerate trials designed to identify predictive biomarkers. Therefore, we set out to comprehensively profile cfDNA samples in mPC, encompassing mHNPC to mCRPC, to gain knowledge relevant for applying ctDNA in a clinical trial context. Although this study was not designed as a predictive or prognostic biomarker study, our data represents a valuable resource for the mPC ctDNA field. We demonstrate the capability of ctDNA-based analysis, but at the same time highlight the challenges which include, but are not limited to, performing analysis where the somatic alterations may originate from multiple independent sources and ctDNA fraction may be low. In addition, we addressed several unanswered research questions of which the key findings are as follows: (1) ctDNA fractions increased gradually from the first to fourth line of therapy. Baseline samples had higher ctDNA fraction compared to follow-up samples, but the difference became non-significant after the second line of mCRPC therapy; (2) in samples with high tumor burden, inactivation of key tumor suppressors was biallelic rather than monoallelic, with only one exception, providing a rationale for assuming a second hit in low ctDNA fraction samples with insufficient sensitivity to detect the second hit; (3) clonal high-impact structural variation is twice as common as point mutations, which challenges the traditional focus on coding regions; (4) the three potentially clinically valuable biomarkers in mPC, microsatellite instability, mutations in genes associated with DNA repair deficiency and *AR* aberrations were detected at expected rates; (5) clonal hematopoiesis occurs frequently, demanding synchronous WBC profiling to avoid false positive variant calls.

Due to the genomic diversity of metastatic cancer, resistance will always arise to single-agent therapies where the duration of response is correlated to the number of cancer cells in a patient [54]. Towards end-stage disease, progression will occur more rapidly, regardless of therapy, with the exception of extreme responders to immunomodulators [14]. Molecular biomarker-driven clinical trials are commonly targeting patients where no approved treatment options remain, although primary outcomes may be hard to achieve if disease burden is too high [55]. Consequently, tumor burden as ctDNA fraction or CTC counts is strongly correlated to conventional outcome measurements [49, 56]. Recently, simple cfDNA concentration estimates were demonstrated to prognosticate patients in a retrospective analysis of two phase III clinical trials [57]. Here, cfDNA concentration was strongly correlated to ctDNA fraction (Additional file 6: Figure S8) suggesting that cfDNA concentration estimation is a surrogate for ctDNA fraction.

Tailored treatment, based on tumor profiling, therefore has the greatest potential early in the course of the disease. Paradoxically, we find that liquid biopsies carry more information towards end-stage disease and currently harbor limited information in a significant fraction of patients starting first- and second-line mCRPC therapy due to low ctDNA fraction (Fig. 1). The tumor burden increased with new systemic (baseline) treatments, from the first to second line and third to fourth. We could not detect a significant difference between second and third baseline samples, likely reflecting the heterogeneity of the treatment sequencing and responses in this all-comers cohort. Comparing the baseline samples, the fraction where it was possible to identify homozygous deletions increased steadily from one third (33/99, before start of first systemic mCRPC treatment) to approximately 50% (59/126, before start of second/third systemic mCRPC treatments) and finally to more than two thirds (11/15, before start of fourth systemic mCRPC treatment). As a consequence, the fraction of patients with BRCA2 homozygous deletion detected here (1.38%, 3/217) was lower than previously reported from two studies analyzing tumor tissue from metastatic patients (2.67%, 4/150 and 4.54%, 5/110) [7, 58]. However, the difference was not statistically significant (two-sided Fisher’s exact test: *p* = 0.450 and *p* = 0.124, respectively).

A potential solution for the low ctDNA fraction samples may be a complementary approach using CTCs to gain insight into ploidy and CNA and ctDNA for mutations and structural rearrangements. However, there are some limitations: we show that CTC counts correlate with ctDNA fraction (Additional file 6: Figure S3), and patients with low ctDNA fraction starting first- and second-line mCRPC therapy, with few exceptions, have low CTC counts (Additional file 6: Figure S9); previous work demonstrates poor success rate (~ 10%) in obtaining high-quality CTC sequencing data from isolated cells [59, 60] necessitating multiple 10-ml blood tubes for CTC analysis in first- and second-line patients. However, recent improvements in harvesting metastatic tissue [32] may provide a fallback if ctDNA profiling fails to identify any relevant biomarkers. As the success rate of harvesting high-quality metastatic tissue is also correlated to tumor burden [29, 30], prospective validation is needed to establish the most feasible approach.

The inherent challenges to complement ctDNA profiling inspired us to investigate the necessity of observing a second hit to infer tumor suppressor deficiency. By deep sequencing of all non-repetitive intronic and exonic regions in *TP53*, *PTEN*, and *RB1* in high ctDNA fraction samples, we investigated whether detection of one clonal high-impact variant is adequate to infer biallelic inactivation. Out of 71 samples in 59 men with ≥ 0.2 ctDNA fraction, 47.5%, 20.3%, and 44.1% harbored biallelic inactivation of *PTEN*, *RB1*, and *TP53*, respectively (Fig. 3). Only one patient carried a clonal high-impact variant, a deletion in *TP53*, without a detectable event on the other allele. These data are encouraging as a large fraction of *TP53* was not possible to sequence due to repetitive DNA (Additional file 6: Figure S6). The observation is consistent with exome sequencing of 150 mCRPC tissues which revealed that biallelic inactivation always had occurred if a high-impact event was observed in a key tumor suppressor such as *PTEN* or *RB1* [7]. Interestingly, residual breakpoints remained in 5/17 samples with a homozygous deletion in *PTEN*, which is detectable, even when tumor burden is low.

The comprehensive profiling of AR surprisingly revealed that 11 out of 85 amplified mCRPC cfDNA samples harbored hotspot mutations in AR. However, concomitant presence of amplification and mutations in a low fraction of cases has previously been described [34, 49]. Speculatively, the multiple existing therapies towards the AR signaling pathway will exert selection pressure differently. This will lead to a complex AR phenotype, where for example an amplified AR is first detectable after first-line androgen deprivation therapy. Subsequent abiraterone treatment may give rise to specific point mutations causing the simultaneous presence of both mutations and amplifications in AR. These speculations are supported by recently published data [49] demonstrating that AR amplifications are not prognostic in the context of abiraterone or enzalutamide treatment, causing a selective pressure on other AR and non-AR alterations as a consequence of therapy.

The advances in targeted sequencing of cancer have rapidly been adopted by multiple companies and transformed into commercially available ctDNA tests [61–63]. Two of these platforms were recently compared with surprisingly low concordance [64]. The lack of accompanying white blood cell germline profiling makes it hard to separate germline variation from somatic [65] and impossible to distinguish clonal hematopoiesis [40–43] from ctDNA unless the ctDNA fraction is high with distinct features of the disease, e.g., the TMPRSS2-ERG gene fusion. In our study, 14.6% of patients harbored clonal expansions in the WBC compartment. Their somatic alterations, detected in germline DNA, were validated in cfDNA from the same individual. However, absolute confirmation of the cell of origin for clonal hematopoiesis events would require investigations involving fluorescence-activated cell sorting of the WBC populations followed by Sanger sequencing or preferably single-cell sequencing, which is beyond the scope of this study. As the targeted sequencing applied here only covered 60 out of 327 driver mutations associated with clonal expansions in the blood [41], the majority of men with mCRPC probably suffer from clonal hematopoiesis. A recent report used digital droplet PCR to investigate hotspot mutations in three genes, commonly mutated in hematopoietic malignancies, in WBC DNA from patients previously profiled with a commercial cfDNA assay [66]. A large fraction of the mutations was detected in the WBC DNA, corroborating our conclusions that to avoid false positive variant calls, sequencing of WBC DNA should be undertaken to the same or higher depth as the cell-free DNA. We therefore discourage the use of commercial assays that only analyze cfDNA from plasma.

Although multiple potentially predictive biomarkers have been reported for metastatic prostate cancer, no level 1 evidence currently exists from prospective randomized clinical trials. Based on our experience to date, we consider ctDNA profiling to be at Technology Readiness Level 7 according to the definition applied in the Horizon 2020 calls [67]. We have therefore initiated a prospective outcome-adaptive, multi-arm, open-label, multiple-assignment randomized biomarker-driven trial in patients with mCRPC where ctDNA profiling will be applied to identify somatic alterations (ProBio, EudraCT Number 2018-002350-78). The goal of the trial is to determine whether treatment choice based on a biomarker signature can improve progression-free survival compared to standard of care in patients with mCRPC and to evaluate the predictive capability of the investigated biomarker signatures.

## Conclusions

This study strengthens the accumulating evidence that ctDNA profiling mirrors the somatic alteration landscape from metastatic tissue by demonstrating, for the first time, that the MSI phenotype may be detected directly from cell-free DNA. To enable acceleration of clinical trials through ctDNA analysis, intronic sequencing of tumor suppressors in combination with synchronous profiling of white blood cells must be applied to prevent inaccurate somatic variant calls, which in turn may reduce the power to identify predictive biomarkers.

## Additional files

Additional file 1: **Supplemental methods.** (DOCX 21 kb)

Additional file 2: **Table S1.** Detailed overview of the capture designs and targeted regions. (XLSX 974 kb)

Additional file 3: **Table S2.** Summary of bioinformatic tools and settings. (XLSX 13 kb)

Additional file 4: **Table S3.** Overview of which samples that were sequenced with which capture design. (XLSX 123 kb)

Additional file 5: **Table S4.** Overview of the number of samples per described analysis. (XLSX 12 kb)

Additional file 6: **Figure S1.** Circulating tumor DNA fraction in baseline samples. **Figure S2.** Tumor burden at different lines of therapy. **Figure S3.** Correlation between circulating tumor cell count and circulating tumor DNA fraction. **Figure S4.** Microsatellite instability by targeted sequencing of microsatellites. **Figure S5.** Chained structural event. **Figure S6.** Gene body panel design. **Figure S7.** Subclonal dynamics. **Figure S8.** Correlation between circulating tumor DNA fraction and cell-free DNA concentration. **Figure S9.** Baseline circulating tumor DNA fraction and circulating tumor cell counts at first- and second-line mCRPC treatment [68, 69]. **Figure S10.** False positive rate evaluation for point mutation variant calling. **Figure S11.** Allele frequencies of structural variants and mutations. (DOCX 3335 kb)

Additional file 7: **Table S5.** CTC count, ctDNA fraction and line of therapy. (XLSX 27 kb)

Additional file 8: **Table S6.** Somatic and germline alterations. (XLSX 141 kb)

Additional file 9: **Table S7.** Table comparing relevant germline alterations, presented here, with other publications. (XLSX 26 kb)

## Abbreviations

AR: Androgen receptor
AR-V7: AR splice variant 7
cfDNA: Cell-free DNA
CNA: Copy-number alteration
CTC: Circulating tumor cell
ctDNA: Circulating tumor DNA
mCRPC: Metastatic castration-resistant prostate cancer
mHNPCs: Metastatic hormone-naïve prostate cancers
mPC: Metastatic prostate cancer
MSI: Microsatellite instability
PARP: Poly (ADP-ribose) polymerase
WBC: White blood cell
