# Supplementary material for: Cell-free DNA profiling of metastatic prostate cancer reveals microsatellite instability, structural rearrangements and clonal hematopoiesis
Source: Genome Med. 2018 Nov 21;10:85. doi: 10.1186/s13073-018-0595-5 (PMC6247769; doi:10.1186/s13073-018-0595-5)
Supplement: Supplementary file 1 — Supplemental methods. (DOCX 21 kb) [file 13073_2018_595_MOESM1_ESM.docx]

**Additional file 1: Supplemental methods**

**Patient cohorts.** Advanced prostate cancer patients were recruited in an all-comer cohort (n=217), ranging from hormone-naïve to castration-resistant, between 2014 and 2017 with histologically confirmed prostate adenocarcinoma (Table 1). Blood samples (n=364) were collected at start of a new line (defined as “baseline” sample) or during a particular systemic therapy (defined as “follow-up” sample). The studied cohort encompassed patients recruited in Stockholm (Prostate Biomarkers (ProBio) cohort, n = 72) and abiraterone or enzalutamide-treated patients recruited in Belgium (CORE-ARV-CTC cohort, n = 145), within the context of a non-interventional clinical study investigating androgen receptor (AR) splice variants in circulating tumor cells (CTCs). Anonymous healthy donor blood (n=36) was collected from healthy men at Hötorgets Blood Centre in central Stockholm. Accounting for both cohorts, cell-free DNA and germline DNA profiling was performed on 217 mPC (metastatic prostate cancer) patients and 36 healthy donors (Table 1). In addition, the FDA-cleared CellSearch CTC technology (Menarini Silicon Biosystems, Italy) was applied for CTC enumeration on 340 out of 364 blood samples processed for circulating tumor DNA (ctDNA) analysis.

**Sample processing and sequencing.** In ProBio, plasma was enriched from 2x10 ml EDTA blood whereas 4-5 ml of blood was available from the CORE-ARV-CTC study. Germline DNA was extracted from leftover EDTA blood after plasma centrifugation. In both studies, the EDTA blood tubes were processed within the same working day, allowing for high-quality ctDNA profiling [[1]](https://paperpile.com/c/oO9wN5/FOUI5). Blood collected in CellSave tubes were shipped to the GZA Sint-Augustinus hospital in Antwerp, to perform CTC counting within 72 hours, as previously described [[2]](https://paperpile.com/c/oO9wN5/VgHQ7). The plasma was stored at -80°C until cell-free DNA (cfDNA) isolation. cfDNA was isolated using the QiaSymphony system (Qiagen, Germany). Purified cfDNA was subjected to fragment analysis for quality control. Germline DNA from white blood cells was isolated using the AllPrep DNA/RNA Micro Kit (Qiagen, Germany). Library prep was mainly performed using the ThruPLEX Plasma-seq kit (Rubicon Genomics, USA). 0.1 – 50 ng of cfDNA and 50 ng of germline DNA was used to create the sequencing libraries. Cell-free DNA profiling was performed with a mix of low-pass whole genome sequencing and targeted sequencing allowing for identification of copy-number alterations (CNAs), small mutations and structural variation. The SeqCap EZ system (Roche Nimblegen, USA) was applied for targeted sequencing. The targeted regions were designed to capture unique regions in the human genome commonly mutated in prostate cancer, identified through literature review. The designs are described in Additional file 2: Table S1. Briefly, the comprehensive designs were aimed at progression samples, targeting common single nucleotide polymorphisms at a certain intervals to enable CNA detection. The smaller designs were tailored for cost efficient deep sequencing in combination with low-pass whole genome sequencing for profiling CNAs. Follow-up samples and samples with <5 ng of cfDNA available were profiled with a smaller, focused design, to enable cost-efficient saturation of the library. Low-pass whole genome sequencing was applied to baseline samples with <5 ng cfDNA to enable CNA analysis.

**Sequence data analysis.** Low-level processing of the sequencing data was performed as previously described [[3]](https://paperpile.com/c/oO9wN5/HqsuQ). Statistical analysis and filtering of variants was conducted in R [[4]](https://paperpile.com/c/oO9wN5/w7yxZ). Bioinformatic tools and settings are summarized in Additional file 3: Table S2. Twenty-one plasma samples displayed significantly increased fraction of discordantly mapped read pairs and low allele frequency variants. Five had previously been profiled from the same blood draw without any quality issues [[3]](https://paperpile.com/c/oO9wN5/HqsuQ). To avoid false positives, these samples were not included for structural variation- or microsatellite instability (MSI) analysis. In addition, mutations below five percent allele fraction were discarded unless they were detected in another sample of the same individual. The data from our previous publications [[1,3]](https://paperpile.com/c/oO9wN5/HqsuQ+FOUI5) was merged with samples that were processed again to increase coverage. Only non-default settings are displayed for the analysis tools below. Germline small variants in blood samples were identified with FreeBayes [[5]](https://paperpile.com/c/oO9wN5/3u1RE) (version: 1.0.1**,** settings: --min-alternate-fraction 0.01 --min-coverage 20, then filtered on QUAL > 5) and annotated with VEP [[6]](https://paperpile.com/c/oO9wN5/mwY55) (version: 83**,** settings: --pick --filter_common --check_alleles --check_existing --total_length --allele_number --no_escape --no_stats --everything --offline). Heterozygous single nucleotide polymorphisms (SNPs) were identified and their allele ratio in the cfDNA samples were used in analysis of copy number alteration and loss of heterozygosity. Downstream analysis of putative oncogenic germline variants was performed in R. Variants were required to be supported by ≥12 reads and ≥20% allele ratio, and to be either annotated as *pathogenic* or *likely pathogenic* in ClinVar [[7]](https://paperpile.com/c/oO9wN5/neKiK), or introduce a premature stop or frameshift in the coding sequence. Findings were inspected manually and annotated for evidence of somatic loss of heterozygosity (LOH) based on the cfDNA copy number profiles and their allele ratio of heterozygous SNPs.

Somatic point mutations in cfDNA samples were identified with VarDict [[8]](https://paperpile.com/c/oO9wN5/AzhKY) **(**settings: -f 0.01 -Q 10, followed by var2vcf_paired.pl with settings -P 0.9 -m 4.25 -M -f 0.01 keeping variants flagged as LikelySomatic or StrongSomatic), using patient-matched blood samples as controls and annotated with VEP (same settings as above). Downstream analysis was performed in R. Known hotspot mutations in *AKT1, APC, AR, ATM, BRAF, CDKN2A, CTNNB1, DICER1, DNAJB1, EGFR, ERBB2, FOXA1, GNAQ, HOXB2, HRAS, IDH1, IDH2, KAT8, KDM6A, KRAS, LRP1, MAP2K1, MED12, PDGFRA, PIK3CA, PIK3CB, PIK3R1, PPP2R1A, PTEN, RAF1, SMAD4, SPOP, TP53* and *XPO1* were not filtered further (≥1% allele frequency). Remaining variants were required to be supported by ≥6 reads and ≥2% allele ratio, and to not be observed ≥2 times in the set of healthy donor cfDNA samples (Additional file 6: Fig. S10). Variants without any effect on coding sequence were also removed except for purity estimation. Remaining variants were reviewed manually and removed if reoccurring in multiple patients at low allele ratio without being a known hotspot mutation. Germline DNA processing failed for four patients (P-GZA3843, P-D6, P-AZSJ044 P-AZSJ055). Healthy donor cfDNA was used as reference to identify point mutations in these patients retaining only high-impact or hotspot mutations. Somatic variants were also annotated for evidence of somatic LOH based on the cfDNA copy number profiles and their allele ratio of heterozygous SNPs. Sublonal variants were pragmatically defined as having an allele frequency < 1/4 of the ctDNA fraction, a similar approach as previously applied for ctDNA in colorectal cancer [[9]](https://paperpile.com/c/oO9wN5/Ow9DA).

Normalized coverage (log_2_-ratio) and segmentation for copy number analysis was produced with qDNAseq [[10]](https://paperpile.com/c/oO9wN5/jCcIM) for low-pass WGS (version: 1.8.0, settings: binSize=15, only using forward reads) and with CNVkit [[11]](https://paperpile.com/c/oO9wN5/1VvfH) for targeted sequence data (version: 0.7.9). The allele ratio of heterozygous SNPs was used with the log-ratio and segmentation to verify and curate observations. Putative somatic focal amplification was called where the median log-ratio at the gene exceeded control regions to the left and right (3-8Mb from gene start or end) by at least 0.5. Putative somatic focal deletion was similarly called where the log-ratio of both control regions exceeded that of the gene by at least 0.3. Amplifications and deletions were curated manually and considered real if supported by the SNP allele ratio and if not attributable to poor data quality such as low coverage and waviness [[12]](https://paperpile.com/c/oO9wN5/iCLKz). Deletions were considered homozygous if no large genomic segments (≥5Mb) had a similar or lower median log-ratio, if the SNP allele ratio of the segment did not indicate allelic imbalance, and if adjacent segments indicated hemizygous deletion or LOH. The sensitivity to detect homozygous deletions and LOH was compromised below 0.2 ctDNA fraction but is also affected by sequence coverage and the clonality of the individual event. Therefore, only samples with ctDNA fraction ≥ 0.2 were used to investigate if one high-impact variant could suffice to infer biallelic inactivation. Somatic deletions indicating *TMPRSS2-ERG* fusion were identified manually from log-ratio and SNP allele ratio. Germline copy number deletion was called where segmented log-ratio of the normal DNA was below -0.5.

The svcaller algorithm was applied for the identification of structural variants as described previously [[3]](https://paperpile.com/c/oO9wN5/HqsuQ). To reduce the proportion of false positive calls the following filters were implemented:

1. Filter out a read pair if there exists an alternative mapping (of equal mapping quality) that is consistent with normal genomic positioning (i.e. pointing towards each other, with an insert size < 1000bp). This eliminates a class of visually apparent false positive calls caused by mapping issues.
2. Reject an event if more than half of the reads have identical start position at either of the termini. This excludes a class of visually apparent false-positive events, in particular frequent weakly supported translocation calls.
3. Filter based on soft-clipped read clustering for duplications or inversions. Exclude the event if the event contains scattered soft-clip regions. An event is considered to have scattered soft-clip regions if the ratio of grouped soft-clips to the number of groups is <= 4. Soft-clippings are first assigned to groups by considering the start and end position of each soft-clippied read and subsequently to the group with the largest soft-clipping count.
4. Filter out events that have overlapping, shared termini: Retain the single largest event, when an overlap occurs.

To improve performance two initial filters were applied:

1. Filter out events where all contributing reads have a mapping quality of zero.
2. Only consider read pairs where both reads map to chromosomes 1-22, X and Y.

Manual evaluation was performed on each variant candidate. Structural variants of unknown significance that occured in multiple germline samples were filtered out. A sample was considered to have a significant structural variants in *AR* if it contained any genomic structural rearrangement that: 1) did not affect exons upstream of cryptic exon 4; 2) affected any exon, including- and downstream of cryptic exon 4. However, non-functional variation such as tandem duplications with the 5’ breakpoint in the ligand binding domain and the 3’ breakpoint downstream of AR, were excluded. In addition, structural events removing *AR* exon 1, known to lead to AR45 expression [[13]](https://paperpile.com/c/oO9wN5/N8boV), were considered to be significant structural variants. All other events in *AR* were considered structural variants of unknown significance. Subclonal point mutations were defined as having an allele frequency < 1/4 of the ctDNA fraction. However, comparing the allele frequencies of structural variants (*PTEN*, *TMPRSS2-ERG* region, *TP53*, *RB1*) to commonly mutated genes in mPC (*AKT1*, *APC*, *ATM*, *BRAF*, *BRCA1*, *BRCA2*, *CDK12*, *CHEK2*, *CTNNB1*, *FOXA1*, *KRAS*, *PIK3CA*, *PTEN*, *RB1*, *SPOP*, *TP53*), in samples that harbored both, revealed consistently lower structural variant allele frequencies (Additional file 6: Fig. S11). The ratio between the median structural variant allele frequency and the median mutation allele frequency was 0.275. Therefore, a structural variant was considered to be subclonal if the allele frequency was < 0.0688 (0.275*0.25) of the ctDNA fraction. After applying this threshold for structural variants on all samples, the number of subclonal structural variants in *ERG*, *PTEN*, *TMPRSS2*, *TP53* and *RB1* was not statistically different from mutations (Number subclonal and clonal for: structural variants, 24 and 162; mutations, 79 and 387. χ2 test: p = 0.201). The current version of the svcaller software is available online: <https://github.com/tomwhi/svcaller>.

Tumor burden (ctDNA fraction, the fraction of cfDNA molecules originating from the cancer cells) was initially estimated from the median allele frequency of somatic point mutations with an allele frequency of at least 5%. However, the variant with highest allele frequency was copy-number adjusted and compared to the median allele frequency. If discordant (+/- 0.05) the ctDNA fraction was corrected. Where somatic copy number alterations suggested a higher tumor burden, it was instead calculated from the observed difference in coverage ratio (2^logR^) associated with gain or loss of one copy (ctDNA fraction = ∆ratio_observed_ / ∆ratio_expected_ where the ∆ratio_expected_ is the difference assuming 100% tumor DNA: 1/2 for a 2N genome, 1/3 for a 3N genome, etc). Only low-fraction structural variation was detected in for P-00030277_3042897, P-00041183_3094920, P-00043867_3124162 and P-NIKO005_20170077. For these four, the average of the number of supporting reads divided by the total read depth at the two breakpoint positions was used as conservative ctDNA fraction estimate.

Evaluation of the sensitivity and specificity to detect MSI was performed on an in-house cohort of 450 colorectal cancer samples out of which 61 had MSI. Microsatellite instability was evaluated by 1) applying the mSINGS [[14]](https://paperpile.com/c/oO9wN5/vJSAd) algorithm 2) comparing the number of mutations, separated into single nucleotide variation and indels. A more stringent threshold was applied (≥10 supporting reads) to filter out artefact variants as intronic and synonymous variants were included. 3) manual inspection of copy-number alteration patterns, where microsatellite unstable tumors have limited copy-number alterations unlike the chromosomal instability phenotype. The MSI colorectal tumors, were diluted in silico to determine a purity- and a fraction unstable microsatellites cutoff for the mSINGS algorithm. Applied on the whole set, a purity cutoff of 10% and and mSINGS 0.1 fraction of unstable microsatellites demonstrated 100% sensitivity and 99% specificity.

Mosaic copy number alterations in blood DNA (smaller effect than consistent with ±1 copy per cell) were considered indications of clonal hematopoiesis. Additional small variants indicating clonal hematopoiesis were identified with VarDict, using patient blood samples with a merged file of all healthy donor blood samples as a control, and annotated with VEP. They were validated in matched cfDNA with VarDict (patient cfDNA with unmatched healthys donor as control). Known hotspot mutations, and other variants supported by ≥6 reads and an allele ratio of 2-25%, with effect on coding sequence, without indication of being SNPs or mapping errors (manual curation) were considered indicative of clonal hematopoiesis.

**References**

[1. Henao Diaz E, Yachnin J, Grönberg H, Lindberg J. The In Vitro Stability of Circulating Tumour DNA. PLoS One. 2016;11:e0168153.](http://paperpile.com/b/oO9wN5/FOUI5)

[2. De Laere B, Oeyen S, Van Oyen P, Ghysel C, Ampe J, Ost P, et al. Circulating tumor cells and survival in abiraterone- and enzalutamide-treated patients with castration-resistant prostate cancer. Prostate. 2018;78:435–45.](http://paperpile.com/b/oO9wN5/VgHQ7)

[3. De Laere B, van Dam P-J, Whitington T, Mayrhofer M, Diaz EH, Van den Eynden G, et al. Comprehensive Profiling of the Androgen Receptor in Liquid Biopsies from Castration-resistant Prostate Cancer Reveals Novel Intra-AR Structural Variation and Splice Variant Expression Patterns. Eur Urol. 2017;72:192–200.](http://paperpile.com/b/oO9wN5/HqsuQ)

[4. Team RC. R: A language and environment for statistical computing. R Foundation for Statistical Computing, Vienna, Austria. 2014. 2016.](http://paperpile.com/b/oO9wN5/w7yxZ)

[5. Garrison E, Marth G. Haplotype-based variant detection from short-read sequencing [Internet]. arXiv [q-bio.GN]. 2012. Available from:](http://paperpile.com/b/oO9wN5/3u1RE) <http://arxiv.org/abs/1207.3907>

[6. McLaren W, Gil L, Hunt SE, Riat HS, Ritchie GRS, Thormann A, et al. The Ensembl Variant Effect Predictor. Genome Biol. 2016;17:122.](http://paperpile.com/b/oO9wN5/mwY55)

[7. Landrum MJ, Lee JM, Riley GR, Jang W, Rubinstein WS, Church DM, et al. ClinVar: public archive of relationships among sequence variation and human phenotype. Nucleic Acids Res. 2014;42:D980–5.](http://paperpile.com/b/oO9wN5/neKiK)

[8. Lai Z, Markovets A, Ahdesmaki M, Chapman B, Hofmann O, McEwen R, et al. VarDict: a novel and versatile variant caller for next-generation sequencing in cancer research. Nucleic Acids Res. 2016;44:e108.](http://paperpile.com/b/oO9wN5/AzhKY)

[9. Strickler JH, Loree JM, Ahronian LG, Parikh AR, Niedzwiecki D, Pereira AAL, et al. Genomic Landscape of Cell-Free DNA in Patients with Colorectal Cancer. Cancer Discov. 2018;8:164–73.](http://paperpile.com/b/oO9wN5/Ow9DA)

[10. Scheinin I, Sie D, Bengtsson H, van de Wiel MA, Olshen AB, van Thuijl HF, et al. DNA copy number analysis of fresh and formalin-fixed specimens by shallow whole-genome sequencing with identification and exclusion of problematic regions in the genome assembly. Genome Res. 2014;24:2022–32.](http://paperpile.com/b/oO9wN5/jCcIM)

[11. Talevich E, Shain AH, Botton T, Bastian BC. CNVkit: Genome-Wide Copy Number Detection and Visualization from Targeted DNA Sequencing. PLoS Comput Biol. 2016;12:e1004873.](http://paperpile.com/b/oO9wN5/1VvfH)

[12. van Heesch S, Mokry M, Boskova V, Junker W, Mehon R, Toonen P, et al. Systematic biases in DNA copy number originate from isolation procedures. Genome Biol. 2013;14:R33.](http://paperpile.com/b/oO9wN5/iCLKz)

[13. Ahrens-Fath I, Politz O, Geserick C, Haendler B. Androgen receptor function is modulated by the tissue-specific AR45 variant. FEBS J. 2005;272:74–84.](http://paperpile.com/b/oO9wN5/N8boV)

[14. Salipante SJ, Scroggins SM, Hampel HL, Turner EH, Pritchard CC. Microsatellite instability detection by next generation sequencing. Clin Chem. 2014;60:1192–9.](http://paperpile.com/b/oO9wN5/vJSAd)
