# Supplementary material for: Cell-free DNA profiling of metastatic prostate cancer reveals microsatellite instability, structural rearrangements and clonal hematopoiesis
Source: Genome Med. 2018 Nov 21;10:85. doi: 10.1186/s13073-018-0595-5 (PMC6247769; doi:10.1186/s13073-018-0595-5)
Supplement: Supplementary file 6 — Figure S1. Circulating tumor DNA fraction in baseline samples. Figure S2. Tumor burden at different lines of therapy. Figure S3. Correlation between circulating tumor cell count and circulating tumor DNA fraction. Figure S4. Microsatellite instability by targeted sequencing of microsatellites. Figure S5. Chained structural event. Figure S6. Gene body panel design. Figure S7. Subclonal dynamics. Figure S8. Correlation between circulating tumor DNA fraction and cell-free DNA concentration. Figure S9. Baseline circulating tumor DNA fraction and circulating tumor cell counts at first- and second-line mCRPC treatment [68, 69]. Figure S10. False positive rate evaluation for point mutation variant calling. Figure S11. Allele frequencies of structural variants and mutations. (DOCX 3335 kb) [file 13073_2018_595_MOESM6_ESM.docx]

**Additional file 6: Supplemental Figures**

**
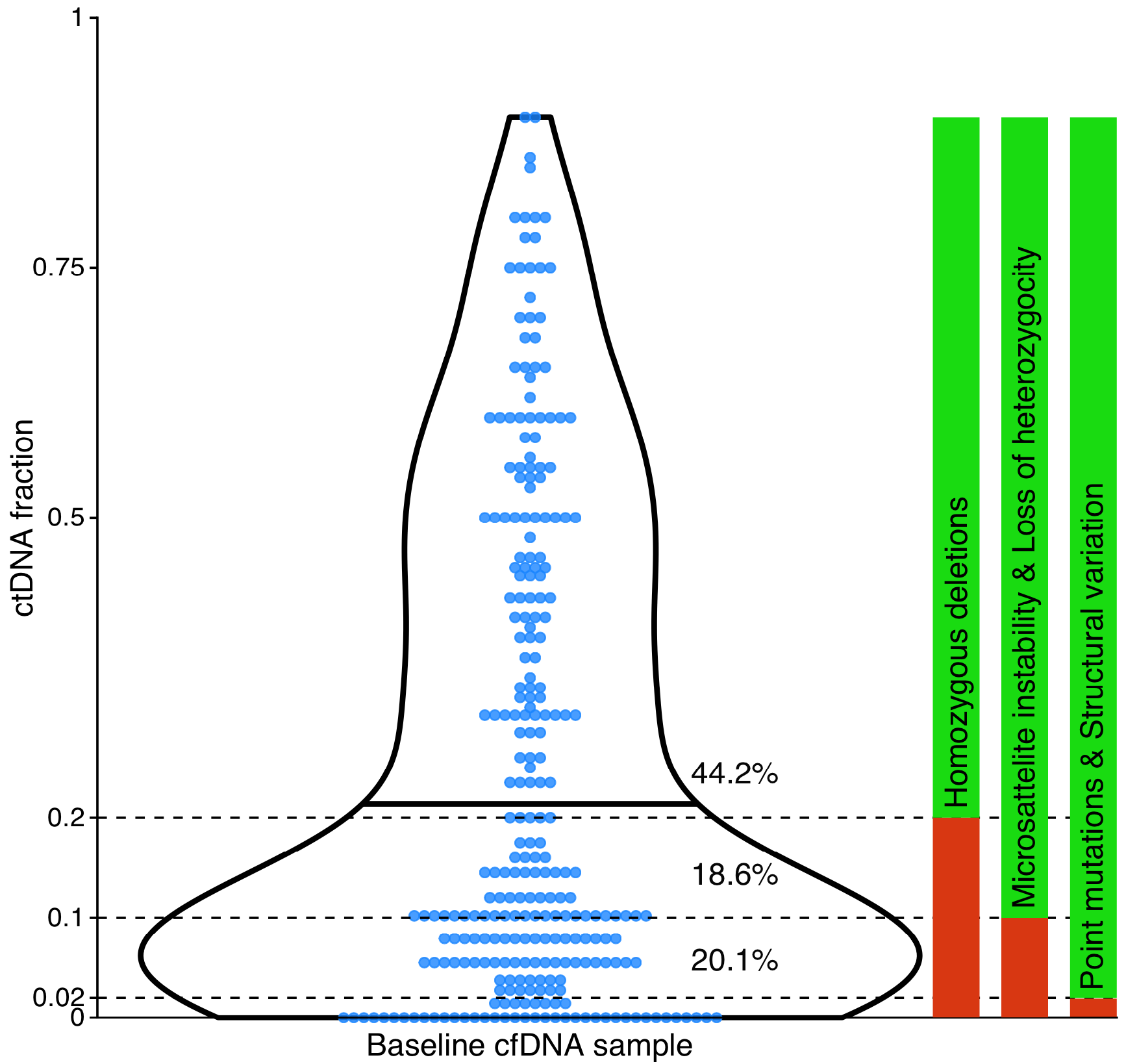
**

**Figure S1 - Circulating tumor DNA fraction in baseline samples.** Violin plot of the circulating tumor DNA fraction in baseline samples (269 samples from 208 unique individuals) defined as blood drawn before the start of a new systemic therapy. The black horizontal line within the violin plot denotes the median of the density estimate. Blue points represent the circulating tumor DNA fraction in individual blood samples. The dashed lines at 0.02, 0.10 and 0.20 denote the cutoffs to reliably detect point mutations, loss of heterozygosity and homozygous deletions, respectively. The percentages denote samples with ≥0.20, <0.20 to ≥0.10 and <0.10 to ≥0.02 circulating tumor DNA fraction. The rightmost green and red bars visualize the percentage of patients were the respective type of alteration is possible to call in relation to the circulating tumor DNA fraction. Y-axis: Circulating tumor DNA fraction. X-axis: Baseline blood samples defined as blood samples collected at start of a new systemic therapy.

**

**Figure S2 - Tumor burden at different lines of therapy. A)** Violin plot of the circulating tumor cell counts per 7.5 ml of blood using the CellSearch platform partitioned according to line of therapy. The black horizontal lines within the violin plots denotes the median of the density estimate. Blue points represent the circulating tumor cell count in individual blood samples. A one-sided Wilcoxon rank sum test was applied to investigate if the baseline samples had higher tumor burden than the follow-up samples. Y-axis: log10 transformed circulating tumor cell counts. X-axis: Line of therapy. **B)** as A but for circulating tumor DNA fraction. In total 364 blood samples are displayed here, however only 340/364 had a successful circulating tumor cell count. The dashed lines at 0.02, 0.10 and 0.20 denote the cutoffs to reliably detect point mutations, loss of heterozygosity and homozygous deletions, respectively. Y-axis: Circulating tumor DNA fraction. Abbreviations: mHNPC[number], metastatic hormone naive prostate cancer and line of therapy; mHSPC[number], metastatic hormone sensitive prostate cancer and line of therapy; mCRPC[number], metastatic castration-resistant prostate cancer and line of therapy; _B, baseline, blood samples collected at start of a new systemic therapy; _F, follow-up, blood samples collected during a systemic therapy; Nbr, number of cell-free DNA samples profiled in each category.

**
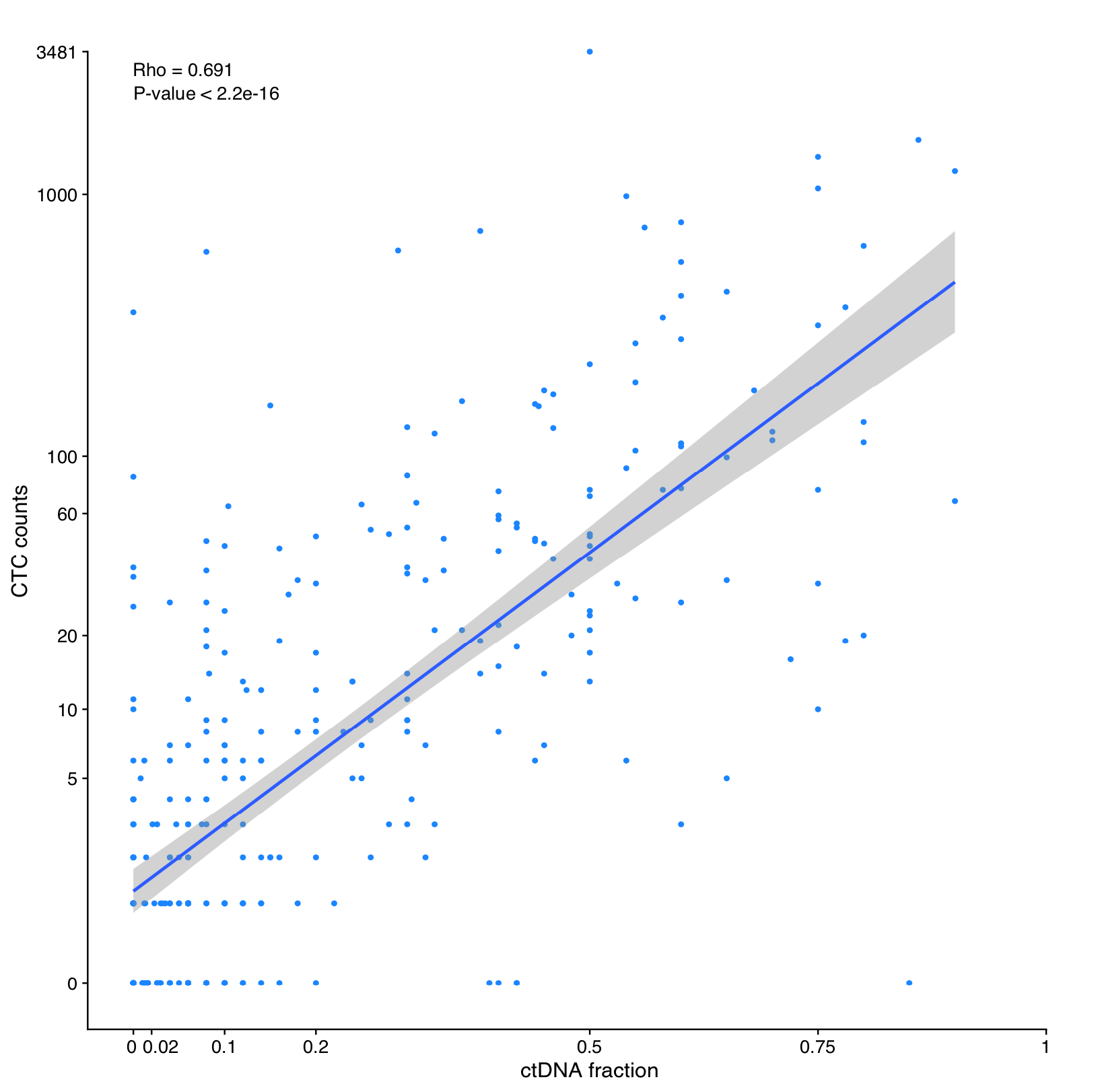
**

**Figure S3 - Correlation between circulating tumor cell count and circulating tumor DNA fraction.** Correlation plot from 340 blood draws with pairwise estimates of number of circulating tumor cells per 7.5 ml of blood and circulating tumor DNA fraction. Y-axis: log10 transformed circulating tumor cell count. X-axis: circulating tumor DNA fraction. Rho; Spearman's correlation coefficient.

**Figure S4 - Microsatellite instability by targeted sequencing of microsatellites.** The mSINGS algorithm[[47]](https://paperpile.com/c/5F4DGA/ONyIG) was evaluated for microsatellite instability detection on low tumor burden cancers using an in-house cohort of 450 colorectal tumor tissues. The whole set of 450 tumor tissues is displayed here at the original tumor purity of each sample. The dashed horizontal line at 0.10 fraction unstable microsatellites denotes the cutoff to reliably detect microsatellite instability. The dashed vertical line at 0.05 median allele frequency was applied as cutoff for purity (equivalent to 0.10 tumor fraction). Y-axis; fraction of unstable microsatellites. X-axis; median allele frequency estimated from single nucleotide variants and indels. The mSINGS pre-set cutoff and our adjusted according to the bottom legend. Abbreviations: microsatellite instability, MSI; microsatellite stable, MSS.

**Figure S5 - Chained structural event.** A chained structural event in patient AZSJ013 connecting intron 2 of TMPRSS2 with chr5, which after 30.1 kb translocates back to intron 3 of ERG, creating a gene-fusion not possible to interrogate via copy-number analysis due to low tumor burden. Reads on chromosome 5; dark yellow. Reads on chromosome 21; dark blue. Soft-clipped sequence support, connecting a read mapped on e.g. chr 21 with chr 5, is colored according to the DNA bases: A, green; T, red; G, blue; C, orange.

**Figure S6 - Gene body panel design.** The targeted regions and the regions possible to capture using Roche Nimblegen oligonucleotides are displayed for A) *TP53*, B) *PTEN* and C) *RB1*. The top tracks PROG_TARGETS represent the regions submitted for design evaluation to Nimblegen. However, as the Nimblegen oligonucleotides are limited in length (average 75bp, range 50-100 bp), repetitive regions, displayed by alignability tracks (green) and repeat tracks (black at the bottom of each panel) are not possible to capture with high accuracy. PROG_CAPTURE denotes the regions passing quality control. This approach allowed us to interrogate 57.6%, 80.0% and 72.2% of the gene body of *TP53*, *PTEN* and *RB1*, respectively.

**Figure S7 - Subclonal dynamics.** Subclonal dynamics for patient **A)** P-GZA4777, **B)** P-KLIN003 and **C)** P−00039325. The top panels display the circulating tumor DNA fraction and the allele frequencies of individuals mutations and structural variants in non-amplified genes. Median allele frequency adjustment was performed on the structural variants with respect to the mutations. Bottom panel for each patient: additional somatic variation colored according to the bottom legend. Genes with multiple somatic alterations of different types (e.g. a point mutation and loss of heterozygosity) are labeled “Multiple alterations”, except for *AR*, were intra-*AR* structural variation and amplifications are kept separate.


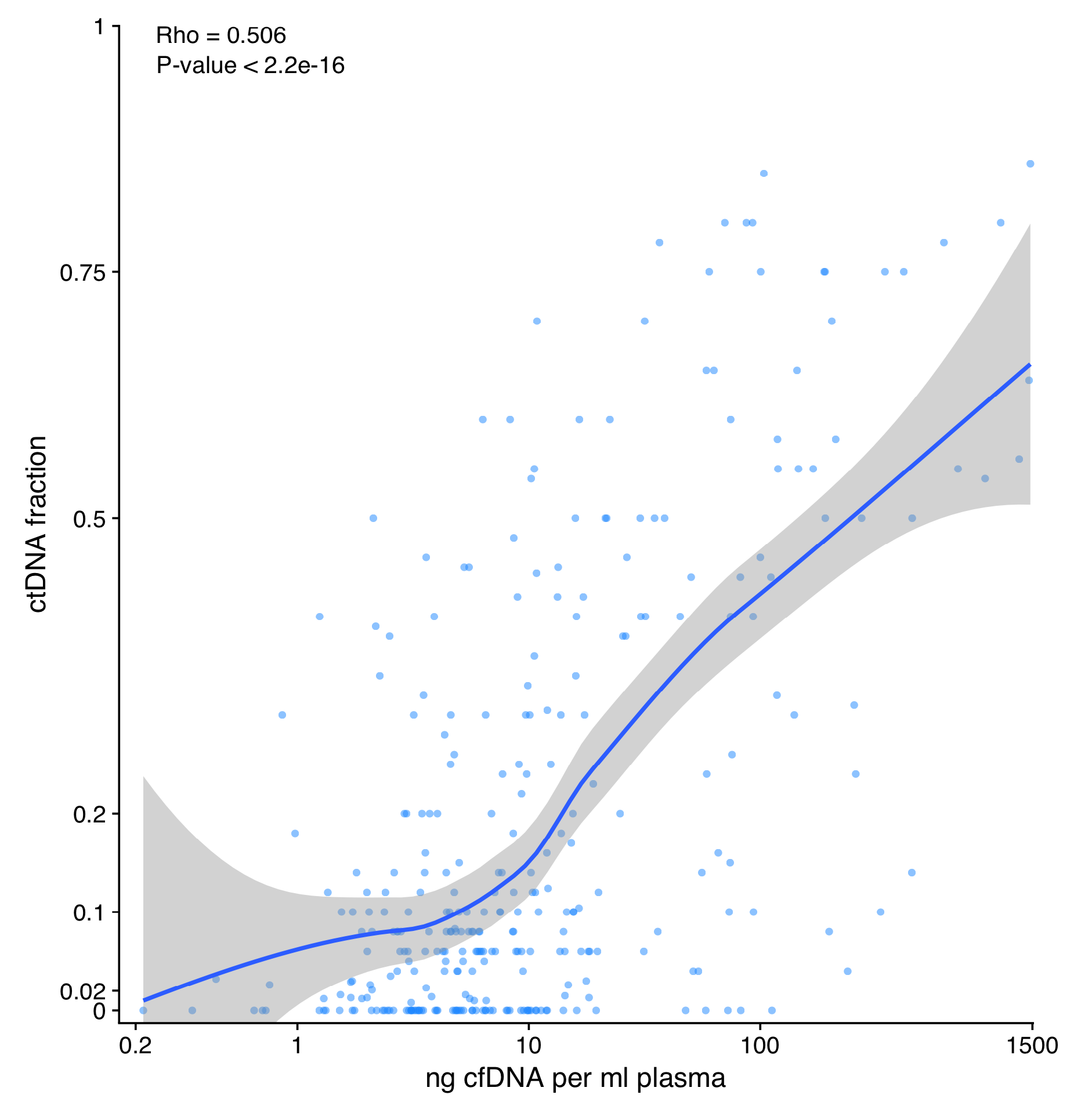


**Figure S8 - Correlation between circulating tumor DNA fraction and cell-free DNA concentration.** Correlation plot from 316 samples with pairwise estimates of circulating tumor DNA fraction and cell-free DNA concentration. Y-axis: Circulating tumor DNA fraction. X-axis: Concentration cell-free DNA as nanogram per milliliter plasma. Rho; Spearman's correlation coefficient.

**Figure S9 - Baseline circulating tumor DNA fraction and circulating tumor cell counts at first and second line mCRPC treatment.** Upper panels, circulating tumor cell counts per 7.5 ml of blood. Y-axis: log10 transformed circulating tumor cell counts. Dashed lines and color indicate: five circulating tumor cells, the prognostic cutoff [[68]](https://paperpile.com/c/5F4DGA/Lovoh); 20 circulating tumor cells, necessary to achieve at least 2-5 high quality copy-number alteration profiles [[69]](https://paperpile.com/c/5F4DGA/JlWU2); 60 circulating tumor cells, needed to obtain multiple high quality profiles for mutational calling [[60]](https://paperpile.com/c/5F4DGA/IX66Y). Bottom panels, circulating tumor DNA fraction. Dashed lines and color indicate: ≥0.02, present cutoff for non-hotspot mutation detection; ≥0.1 cutoff to detect loss of heterozygosity; ≥0.2 cutoff to identify clonal homozygous deletions. X-axis: Line of therapy. Abbreviations: mCRPC1_B, first line metastatic castration-resistant prostate cancer baseline sample; mCRPC2_B, second line metastatic castration-resistant prostate cancer baseline sample.

**Figure S10 - False positive rate evaluation for point mutation variant calling.** Sequence coverage and variant allele frequency of putative somatic variants observed in 18 healthy donor cell-free DNA samples. Of putative variants that exceed 2% allele ratio and 6 alternative allele reads, 10 would affect coding sequence, indicating an average false positive rate of less than one variant per sample although none occurred in any clinically relevant gene.

**Figure S11 - Allele frequencies of structural variants and mutations.** The allele frequencies were compared between structural variants (*PTEN*, *RB1*, *TMPRSS2-ERG* gene fusion, *TP53*) and somatic mutations in key genes commonly mutated in metastatic prostate cancer (*AKT1*, *APC*, *ATM*, *BRAF*, *BRCA1*, *BRCA2*, *CDK12*, *CHEK2*, *CTNNB1*, *FOXA1*, *KRAS*, *PIK3CA*, *PTEN*, *RB1*, *SPOP*, *TP53*). Only individuals that both harbored structural variation and mutations were used for this comparison. In total 137 structural variants were compared to 97 mutations. Only the ends that overlap targeted positions in the genome were used to calculate the allele frequency for the structural variants. Each mutation is plotted versus all structural variants from the same sample. Structural variants are annotated according to the bottom legend: One end on target, structural variants with only one end overlapping targeted regions; Two ends on target, structural variants where both ends overlap targeted regions; Max allele frequency, the end of an individual structural variant with maximum allele frequency; Min allele frequency, the end of an individual structural variant with minimum allele frequency.
